# Brain Network and Abnormal Hemispheric Asymmetry Analyses to Explore the Marginal Differences in Glucose Metabolic Distributions Among Alzheimer's Disease, Parkinson's Disease Dementia, and Lewy Body Dementia

**DOI:** 10.3389/fneur.2019.00369

**Published:** 2019-04-12

**Authors:** Danyan Chen, Jiehui Jiang, Jiaying Lu, Ping Wu, Huiwei Zhang, Chuantao Zuo, Kuangyu Shi

**Affiliations:** ^1^Shanghai Institute for Advanced Communication and Data Science, Shanghai University, Shanghai, China; ^2^Key Laboratory of Specialty Fiber Optics and Optical Access Networks, Joint International Research Laboratory of Specialty Fiber Optics and Advanced Communication, Shanghai University, Shanghai, China; ^3^PET Center, Huashan Hospital, Fudan University, Shanghai, China; ^4^Institute of Functional and Molecular Medical Imaging, Fudan University, Shanghai, China; ^5^Department Nuclear Medicine, University of Bern, Bern, Switzerland; ^6^Department of Informatics, Technical University of Munich, Munich, Germany

**Keywords:** dementia, FDG-PET imaging, brain network, graph theory, asymmetry

## Abstract

Facilitating accurate diagnosis and ensuring appropriate treatment of dementia subtypes, including Alzheimer's disease (AD), Parkinson's disease dementia (PDD), and Lewy body dementia (DLB), is clinically important. However, the differences in glucose metabolic distribution among these three dementia subtypes are minor, which can result in difficulties in diagnosis by visual assessment or traditional quantification methods. Here, we explored this issue using novel approaches, including brain network and abnormal hemispheric asymmetry analyses. We generated 18F-labeled fluorodeoxyglucose (18F-FDG) positron emission tomography (PET) images from patients with AD, PDD, and DLB, and healthy control (HC) subjects (*n* = 22, 18, 22, and 22, respectively) from Huashan hospital, Shanghai, China. Brain network properties were measured and between-group differences evaluated using graph theory. We also calculated and explored asymmetry indices for the cerebral hemispheres in the four groups, to explore whether differences between the two hemispheres were characteristic of each group. Our study revealed significant differences in the network properties of the HC and AD groups (small-world coefficient, 1.36 vs. 1.28; clustering coefficient, 1.48 vs. 1.59; characteristic path length, 1.57 vs. 1.64). In addition, differing hub regions were identified in the different dementias. We also identified rightward asymmetry in the hemispheric brain networks of patients with AD and DLB, and leftward asymmetry in the hemispheric brain networks of patients with PDD, which were attributable to aberrant topological properties in the corresponding hemispheres.

## Introduction

Dementia is progressive cognitive deterioration caused by brain injury or disease. The deterioration is much more rapid than that associated with normal aging, and affects memory, attention, language, and problem-solving skills (1, 2). The etiology of dementia can be categorized as Alzheimer's disease (AD), Parkinson's disease dementia (PDD), Lewy body dementia (DLB), vascular dementia, and other dementias.

The technique, 18F-labeled fluorodeoxyglucose (18F-FDG) positron emission tomography (PET), which reveals glucose metabolic distribution across the whole brain, is the most commonly used, accurate and effective, gold-standard method for diagnosis of early-stage dementia. Based on quantitative analysis of FDG-PET scans, scholars have explored the clinically significant differences between healthy controls and patients with different dementia subtypes, including AD (3), PDD (4), and DLB (5); however, differences in the glucose metabolic distributions underlying the various dementia subtypes in their early stages are usually slight, representing a challenge for clinicians in distinguishing among dementia subtypes by visual assessment or traditional quantification methods (4, 6, 7). Hence, novel methods are required to facilitate accurate diagnosis and ensure appropriate treatment for patients with dementia.

Recently, analyses of brain networks and abnormal hemispheric asymmetry have been considered as alternative neuroimaging approaches for exploration of marginal differences in patients with dementia (8–10). In brain network analysis, graph theory has been widely applied to study glucose metabolic transformation in different brain regions in patients with dementia. For example, Caminiti et al. studied 42 patients with DLB and 42 healthy controls, using sparse inverse covariance estimation and graph theory. They detected substantial alterations in connectivity indices, brain modularity, and hub configurations. Further, they reported observed decreases in local metabolic connectivity within the occipital cortex, thalamus, and cerebellum, and increases in the frontal, temporal, parietal, and basal ganglia regions (6). In addition, there are long-range disconnection among these brain regions, supporting disruption of the functional hierarchy that characterizes the healthy brain (11).

Furthermore, analyses of abnormal hemispheric asymmetry, based on brain network parameters that have long been measured and compared, have also been used to study dementia. For example, cortical volume (12, 13), cortical surface area (13, 14), and other asymmetries, can be evaluated to distinguish dementia subtypes. Functional connection differences, in terms of brain network metabolic efficiencies (9), also indicate abnormal hemispheric asymmetry in patients with AD and mild cognitive impairment (MCI). The results show that in AD dementia, left hemisphere degeneration is more rapid, and the damage more severe, as shown by decreases in patient nerve fiber bundle fractional anisotropy (FA) (15, 16) and impaired network efficiency (9). Additionally, Gilman used PET with [11C] dihydrotetrabenazine to examine striatal monoaminergic presynaptic terminal density in patients with DLB and AD. The DLB and AD groups showed significant binding asymmetry in the posterior putamen (17). Walter et al. used asymmetry indices based on transcranial sonography to successfully discriminate PDD from DLB (18). Nevertheless, no study to date has directly compared abnormal hemispheric asymmetry detected using FDG-PET imaging among patients with AD, PDD, and DLB.

This study therefore recruited patients with AD, PDD, and DLB, in comparison with HC, with two main objectives: (1) exploration of the disrupted glucose metabolism network topology (brain network) and comparison of related parameters, and (2) exploration of hemispheric asymmetry.

## Materials and Methods

### Participants

Metabolic brain images were acquired using 18F-labeled fluorodeoxyglucose (18F-FDG) positron emission tomography (PET) from four groups, including 22 healthy subjects, and 22, 18, and 22 patients with AD, PDD, and DLB, respectively. Subjects were recruited from the PET Center of Huashan Hospital, Shanghai, China. All participants were right-handed. Three days before and after PET image acquisition, we obtained basic information about these subjects, including their age, sex, and Mini-Mental State Examination (MMSE) scores (Table 1). All aspects of the study were approved by the Human Studies Institutional Review Board, Huashan Hospital.

**Table 1:** Statistical information from all participants.

**Info**	**HC(*n* = 22)**	**AD(*n* = 22)**	**PDD(*n* = 18)**	**DLB(*n* = 22)**	***p*-value**
Male: female	5:17	16:6	12:6	21:1	*p* < 0.001^a^
Age	63.5 ± 5.6	57.3 ± 6.4	63.5 ± 7.4	66.9 ± 8.4	*p* = 0.21^b^
MMSE	28.9 ± 1.3	20.8 ± 4.2	23.9 ± 5.3	20.0 ± 5.0	*p* < 0.001^b^

Age and MMSE are presented as mean ± standard deviation.

a χ^2^ test, HC, AD, PDD, and DLB.

b Analysis of variance HC, AD, PDD, and DLB.

*MMSE, Mini-Mental State Examination*.

### PET Image Acquisition and Preprocessing

Whole brain PET images were acquired from 84 participants using a Siemens Biograph 64 PET/CT machine in the PET Center of Huashan Hospital in Shanghai, China. The spatial resolution of the PET scanner was 5.9 mm full-width at half-maximum (FWHM) in the transaxial plane and 5.5 mm FWHM in the axial plane. All subjects were intravenously injected with 185 MBq FDG in a dimly-lit, quiet room. They were asked to keep their eyes closed for 1 h to reduce possible activities which could obscure the results. Thereafter, static emission scans were conducted for 10 min. Using a Shepp–Logan filter, we implemented a filtered back projection algorithm to reconstruct transaxial images with the following dimensions: 168 × 168 × 148 matrices and a size of 2.0 × 2.0 × 1.5 mm.

All original images were obtained in Digital Imaging and Communications in Medicine format and converted to NIfTI format using DCM2NII software (https://www.nitrc.org/projects/dcm2nii/). For pre-processing of converted images, Statistical Parametric Mapping 12 software (Department of Imaging Neuroscience, Institute of Neurology, London, United Kingdom) was implemented in MATLAB (2016)^1^ (Mathworks Inc, Sherborn, MA, United States). First, PET images were spatially normalized to Montreal Neurological Institute (McGill University, Montreal, Canada) space. In this step, we use SPM software to spatially register each image separately to the reference PET template. Spatial registration was a completely automated procedure, based on 12-parameter affine transformation. Then, normalized images were smoothed by convolution, using an isotropic Gaussian kernel with 10 × 10 × 10 mm^3^ FWHM. Finally, images were converted to grayscale, with 256 gray levels.

### Brain Network Construction

Brain function networks were constructed for the HC, AD, PDD, and DLB groups using a graph theory approach. First, using a brain template to cover brain tissue, we chose Standardized Automated Anatomical Labeling (AAL) template (the part that removes the cerebellum, using only 90 regions of the brain). Secondly, the value of each network node was calculated. The globally normalization was obtained by averaging the intensity values of the ROI in each patient, calculating the correlation matrix between the nodes in the group to obtain the correlation matrix. Partial correlation coefficients were used here to exclude age and sex interference. Finally, the sparsity threshold (n) method was used to determine whether the connection is taken into account. The connection strength which is higher than top n% in the matrix was counted as 1, and vice versa. In this way, the aforementioned correlation coefficient matrix can be converted into a set of binarization matrices with a threshold of 6–40% (19–22).

### Brain Network Analysis

After network construction, brain function networks were calculated for the HC, AD, PDD, and DLB groups, using a graph theory approach. The following network parameters were calculated: clustering coefficient (C), characteristic path length (L), gamma, lambda, small-world coefficient (sigma), local efficiency (localE), global efficiency (globalE), and node betweenness centrality (BC). In graph theory, the C of a network is as a measure of the degree to which nodes in a graph tend to cluster together, while L is as a measure of the efficiency of the information, or the mass transport, of a network. A small-world network should meet the following criteria: gamma >> 1, lambda ≈ 1, and sigma > 1. GlobalE and localE are measures of the efficiency of information exchange in an entire network and a local network, respectively. BC is typically used to determine the number of candidate hubs in a network. According to previous studies, nodes with high bi (BC/averaged BC) values (bi > 1.5) were considered candidate hubs. We further differentiated the AAL template according to the left and right hemispheres, and constructed brain networks for both hemispheres (including 45 brain regions), to further evaluate the efficiencies of the hemispheres. In this study, network characteristic parameters were calculated using the open source graph analysis software, graph theoretical network analysis (GRETNA) (23) and The Brain Connectivity Toolbox (https://sites.google.com/site/bctnet/).

To determine the statistical significance of differences in network parameters in the AD, PDD, DLB, and HC groups, we used a non-parametric permutation test with 1,000 repetitions.

### Seed-Based Correlation Analysis

After determining hubs in the four groups, brain areas that were significantly changed, or isolated, among different groups were subjected to further analysis. Seed-based correlation analysis was used to further explore the details of the connectivity between other brain regions and those that were significantly altered.

First, the Pearson correlation coefficient was calculated for each voxel across the whole brain in a designated altered brain region, and the obtained correlation coefficients converted to z-values using Fisher's r-to-z transformation, to ensure that they obeyed an approximate Gaussian distribution, using the following formula:

$$\begin{array}{l} z_{i}=1/2\times \text{log}\left[\left(1+r_{i}\right)/\left(1-r_{i}\right)\right] \end{array}$$

where *r*_*i*_ refers to the correlation coefficients, and *z*_*i*_ the transformed z-values. Finally, these z-values were compared among groups using Z statistics with the formula:

$$\begin{array}{l} \text{z}=\left(z_{1}-z_{2}\right)/\sqrt{1/\left({\text{n}}_{1}-3\right)+1/\left({\text{n}}_{2}-3\right)} \end{array}$$

where n_1_ and n_2_ refer to the samples of two groups (24). The false discovery rate (FDR) procedure was performed, at a *P* value of 0.05, to adjust for multiple comparisons (3).

### Within-Group Asymmetry

Numerous studies have revealed diverse aspects of variation in different brain networks in patients with brain diseases. The common feature of these investigations is that the brain networks of the patients exhibit different degrees of degradation of small world characteristics (25, 26). Degradation of the small world characteristics of a brain network indicates reduction in its global and local information processing efficiencies. Therefore, we focused on calculation of asymmetries in the efficiency of brain networks. To determine the statistical significance of asymmetry indices in the AD, PDD, DLB, and HC groups, we used a random sampling permutation test with 1,000 repetitions (27). A two-sample t-test was used to evaluate differences between each disease group and the HC group, to determine if they were significant.

In addition, to assess the degree of differences in left and right hemispheric networks, AI values were computed for each left-right pair, using the following formula (8, 10, 28):

$$\begin{array}{l} \text{AI}=200^{*}|\frac{M_{R}-M_{L}}{M_{R}+M_{L}}| \end{array}$$

where *M*_*R*_ and *M*_*L*_ represent the global and local network efficiencies of the right and left hemispheric networks, respectively.

## Results

### Network Parameters

**Figure 1:**
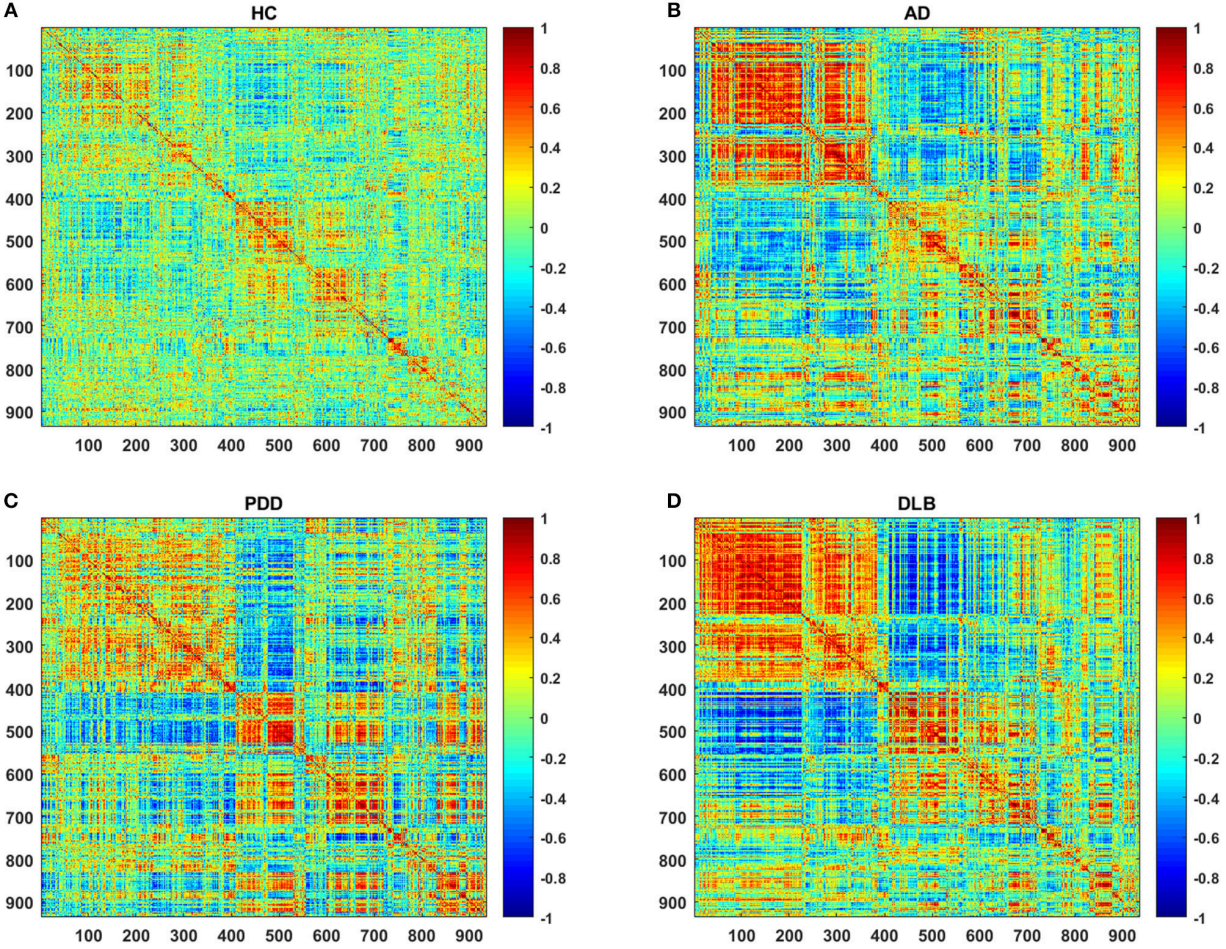
Partial correlation coefficient matrices (indicated by the color bar, ranging from −1.0 to 1.0) for the **(A)** HC, **(B)** AD, **(C)** PDD, and **(D)** DLB groups.

**Figure 2:**
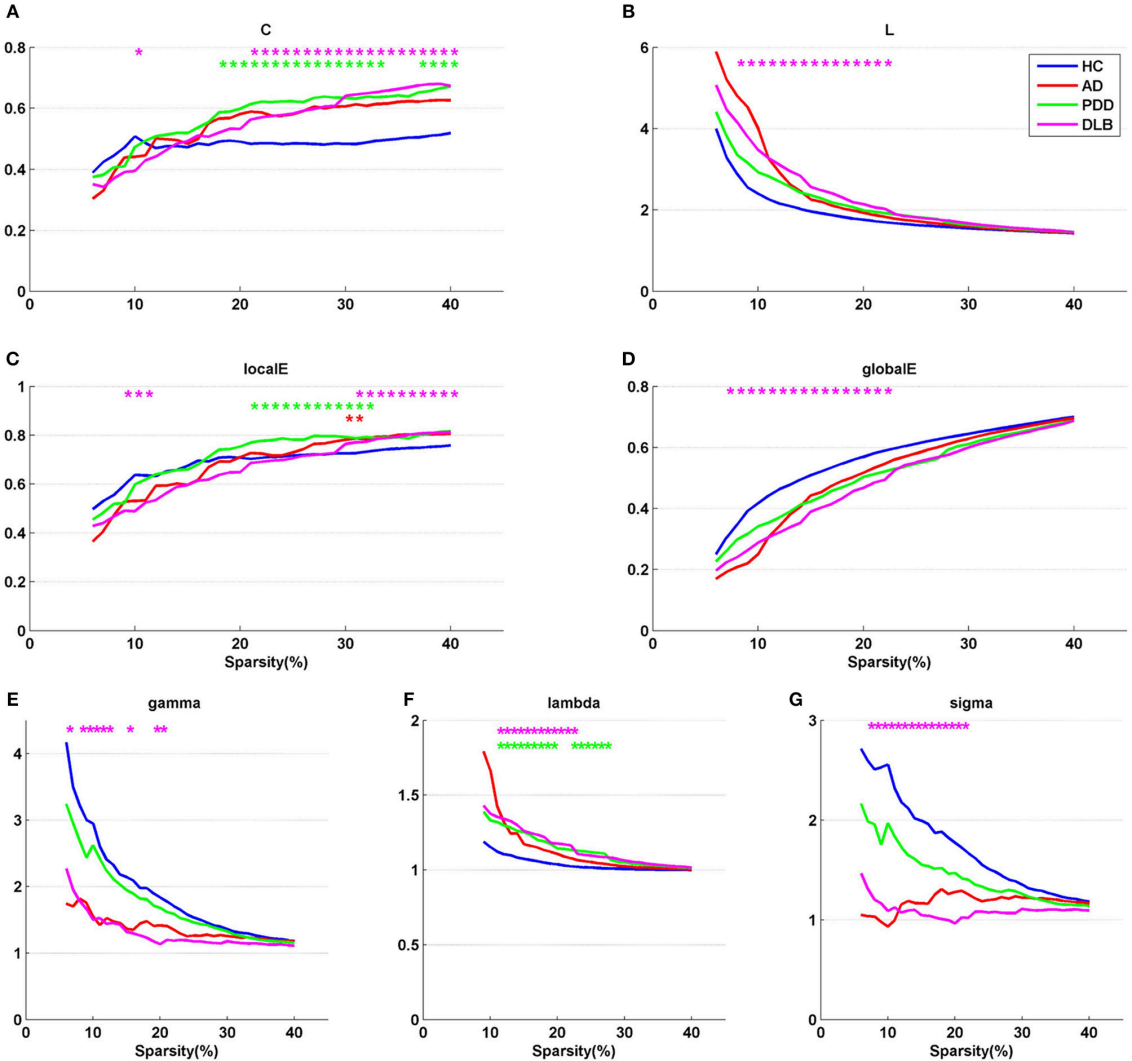
Network parameters, **(A)** C, **(B)** L, **(C)** localE, **(D)** globalE, **(E)** gamma, **(F)** lambda, and **(G)** sigma, for the four groups. X axis coordinates represents sparsity threshold values, ranging from 6 to 40%. Blue curve, HC group; red curve, AD group; green curve, PDD group; magenta curve, DLB group. Red, green, and magenta asterisks represent significant differences in sparsity threshold between HC vs. AD, HC vs. PDD, and HC vs. DLB groups, respectively (*p* < 0.05).

Figure 1 shows partial correlation coefficient matrices for the four groups, produced by partial correlation analysis. The data clearly generate visually divergent color distributions in the different groups (i.e., the groups exhibit divergent partial correlation coefficients). The various network parameters for the four groups are presented in Figure 2. C, L, localE, globalE, gamma, lambda, and sigma values were calculated separately for each of the four groups, within a range of sparsity from 6–40%.

The results demonstrate that all four groups fulfilled the criteria gamma >> 1, lambda ~1, and sigma > 1 in a sparsity range of 6–40%, indicating that all groups exhibited small-world properties. The characteristics of the small-world attributes of the four groups are presented in Figures 2E–G. Compared with the HC group, the three dementia groups showed a loss of small-world network characteristics, with the most marked difference in the DLB group. For example, the mean value of sigma in the sparsity range 6–40% was 1.73 in the HC group, 1.45 in the AD group, and 1.18 in the PDD group, while it was only 1.09 in the DLB group. Compared with the HC group, the clustering coefficients of the AD, PDD, and DLB groups were higher, and the characteristic path lengths were longer (Figure 2A). The values of L in the three dementia groups were also greater than that of the HC group (Figure 2B). Local and global efficiency values, were lowest in the DLB group, indicating that the efficiencies of local information processing, global communication efficiency across the network, and integration of information between the different regions of the brain, were lowest in this group.

A non-parametric permutation test was applied to test the statistical significance of between-group differences among the AD, PDD, DLB, and HC groups (*p* < 0.05). Compared with the HC group, all network parameters in the DLB group were significantly different at specific sparsity values. LocalE was significantly higher in the AD group than the HC group at sparsity values of 30–31%; C was significantly higher in the PDD group than the HC group sparsity values of 18–33% and 37–40%; localE was significantly higher in the PDD group than the HC group at sparsity values of 21–32%; while lambda was significantly higher in the PDD group than the HC group at sparsity values of 11–19% and 22–27%.

### Hub Regions

Normalized betweenness centrality (bi) is a very useful indicator in graph-based theory because it reflects the relative importance of nodes in the network, helping us to identify the hub nodes. Before determining the candidate hubs in the four network, we first chose a reasonable sparsity. This sparsity should ensure that all four networks can be fully connected, without missing any brain region, biologically reasonable, and should be as small as possible to reflect the differences between the four groups. In this experiment, we chose 28%.

**Figure 3:**
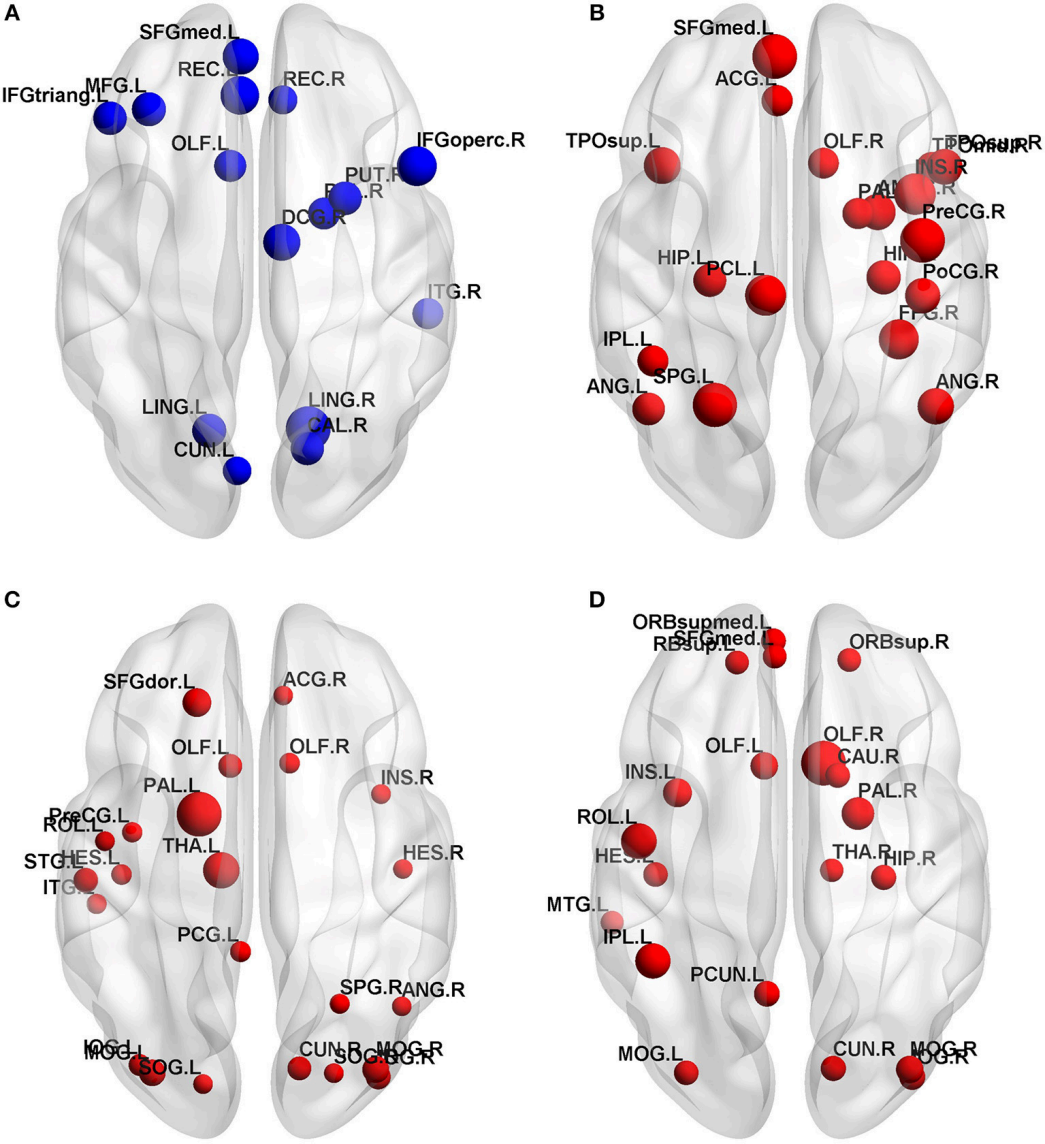
Hub nodes in the **(A)** HC, **(B)** AD, **(C)** PDD, and **(D)** DLB groups. Node sizes are proportional to values of normalized centrality.

In the HC, AD, PDD and DLB groups, 15, 19, 23, and 20 hub nodes appeared respectively on the principle of bi>1.5. We showed its distribution and importance in an axial view in Figure 3. In general, the four groups were mainly located in the association area. Anatomically, the prefrontal and occipital cortex contained most hubs in the HC group; the temporal and parietal cortex were important for the AD hub; the hubs of the PDD group were mainly located in the occipital and temporal cortex; DLB was more dispersed, with hubs distributed in the prefrontal, occipital and subcortical cortex. And the importance of the temporal cortex was reduced in the PDD and DLB groups compared to the AD group. All bi values for each of the four groups are listed in Appendix A.

### Seed Correlation Analysis

To further investigate the detailed connectivity associated with the hubs in the four groups, the right middle temporal gyrus (MTG.R) was selected as a seed. This region was selected for two primary reasons: first, the MTG.R was a hub node in the HC, PDD, and DLB groups (bi > 1.5) and also relatively important in the AD group (bi = 1.48); second, in various previous studies, the MTG.R has been identified as having a very important role in dementia pathogenesis (29–31).

**Figure 4:**
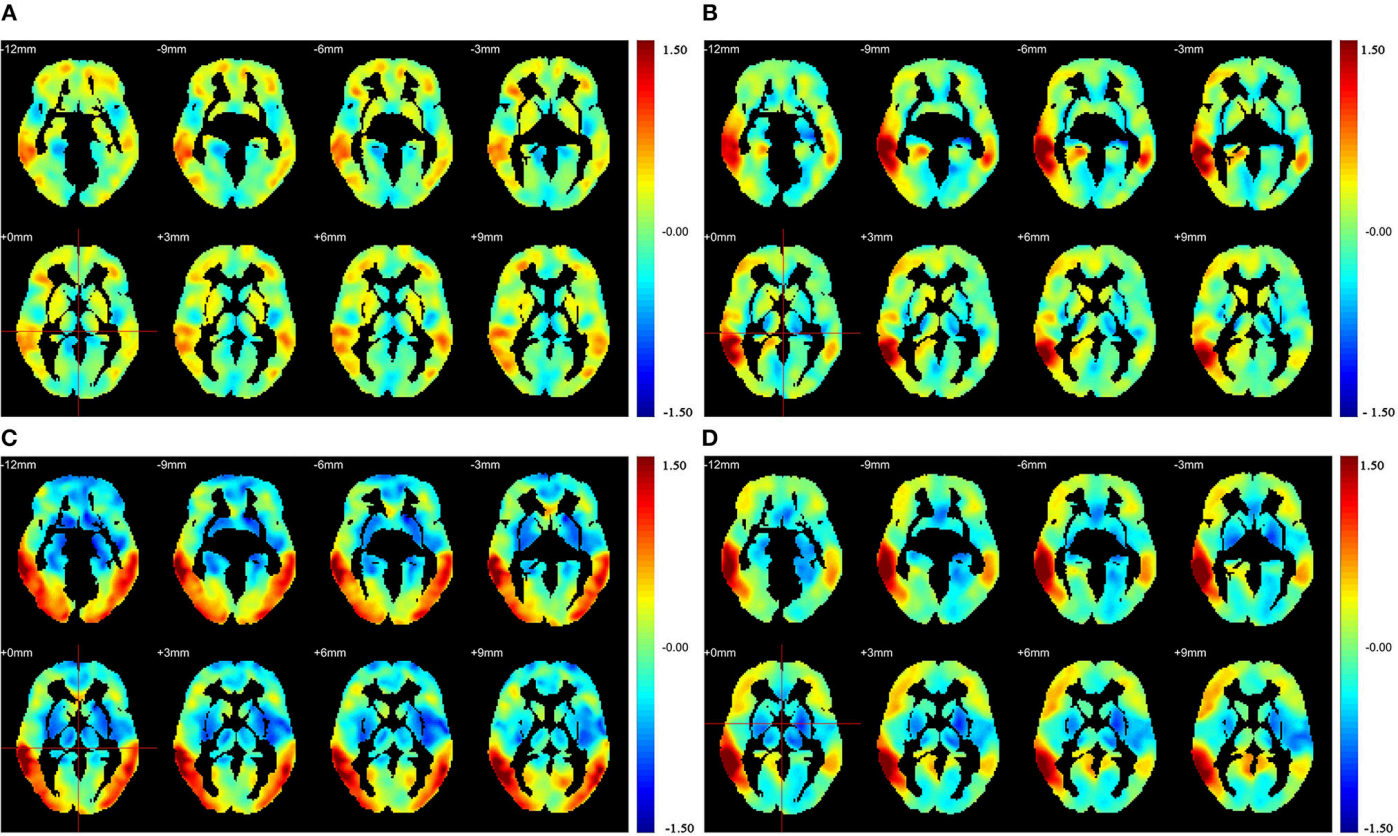
Results of seed correlation analysis for the **(A)** HC, **(B)** AD, **(C)** PDD, and **(D)** DLB groups. Images were drawn using the REST toolbox (32).

Figure 4 illustrates the correlation coefficient maps (R-maps) associated with the MTG.R in the HC, AD, PDD, and DLB groups. In the HC group, the R-map showed that superior temporal gyrus and middle frontal gyrus had strengthened connections with the MTG.R, while the paracentral lobule and lingual gyrus had weakened connections. In the AD group, strengthened connections with the MTG.R were focused on the superior temporal gyrus and angular gyrus, while regions with weakened connections were primarily in the paracentral lobule and supplementary motor areas. In the PDD group, strengthened connections with the MTG.R were focused on the precuneus and angular gyrus, while weakened connection regions were in the precentral gyrus and supplementary motor areas. In the DLB group, strengthened connections with the MTG.R were primarily in the middle frontal gyrus and precuneus, while regions with weaker connections were mainly in the fusiform gyrus and parietal gyrus.

**Figure 5:**
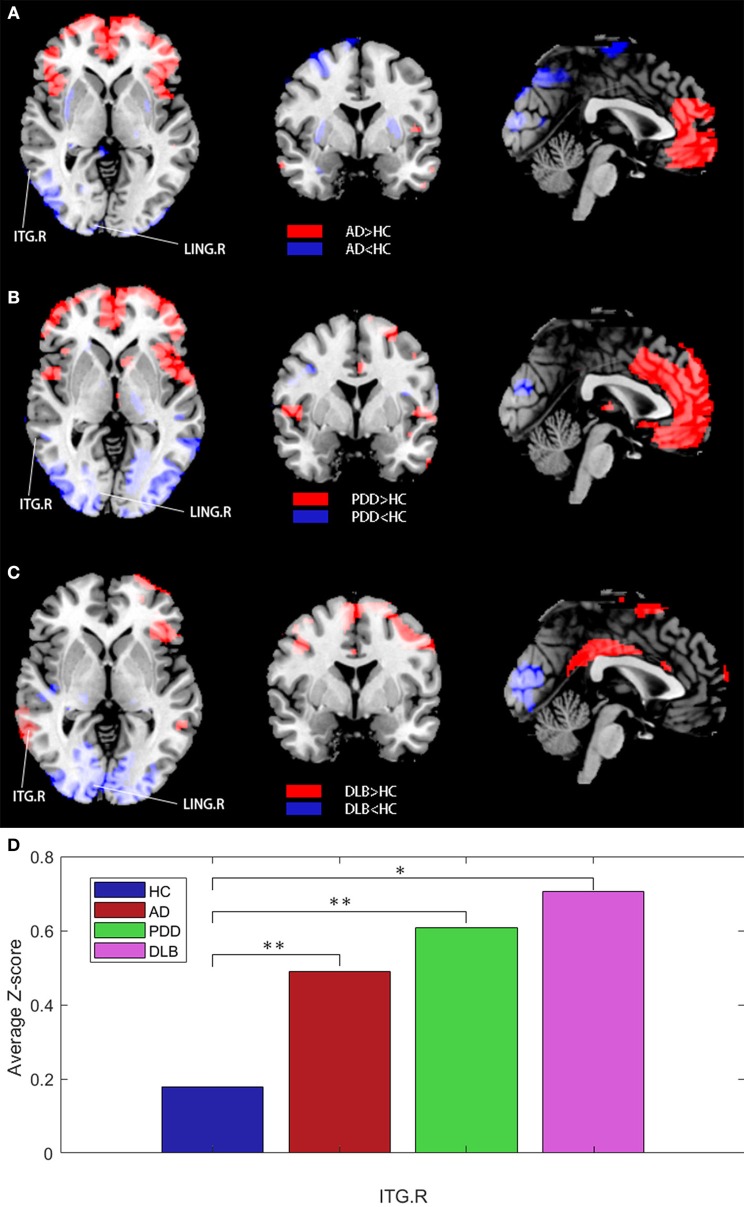
**(A–C)** *Z*-statistics map showing the brain regions with strengthened or weakened connections with the right middle temporal gyrus in patient groups compared with the HC group (FDR corrected *P* < 0.05). Red, strengthened; blue, weakened. **(D)** Average *Z*-scores for the ITG.R in the four groups (^*^P < 0.05, ^**^P > 0.05).

Next, all patient data were used as a reference for further analysis. Z-statistical mapping was performed for the MTG.R regions of the four groups. Figure 5 illustrates the results from the DLB group, obtained using the Z-statistical test (z-map). Compared with the HC group, the results of Z-statistical analysis indicated brain regions with established connections with the MTG.R in the DLB group. These regions were in the prefrontal cortex, including the right inferior temporal gyrus (ITG.R), the right angular gyrus (ANG.R), and the right temporal pole:middle temporal gyrus (TPOmid.R). The right lingual gyrus (LING.R) and some occipital lobe regions also had weakened connections with the MTG.R (FDR corrected *P* < 0.05). To represent the results more clearly, we plotted the average *Z*-scores of the significant connections in the enhanced ITG.R brain region in the four groups. The ITG.R region in the DLB group clearly had the strongest metabolic activity connection, relative to the HC group.

The same analysis was also performed for the AD and PDD groups. For the AD group, connection enhancements were relatively scattered, mainly in the right middle occipital gyrus (MOG.R) and right inferior parietal, but also in the supramarginal and angular gyri (IPL.R), and the right precuneus (PCUN.R). These brain regions overlap with the brain default mode network (DMN) to a large extent. For the PDD group, connection enhancements were mainly located in the parietal region, including the bilateral postcentral gyrus (PoCG.L and PoCG.R); brain regions with weakened connections were mainly the left precentral gyrus (PreCG.L) and the left gyrus rectus (REC.L).

### Within-Group Asymmetry of Network Efficiencies

**Figure 6:**
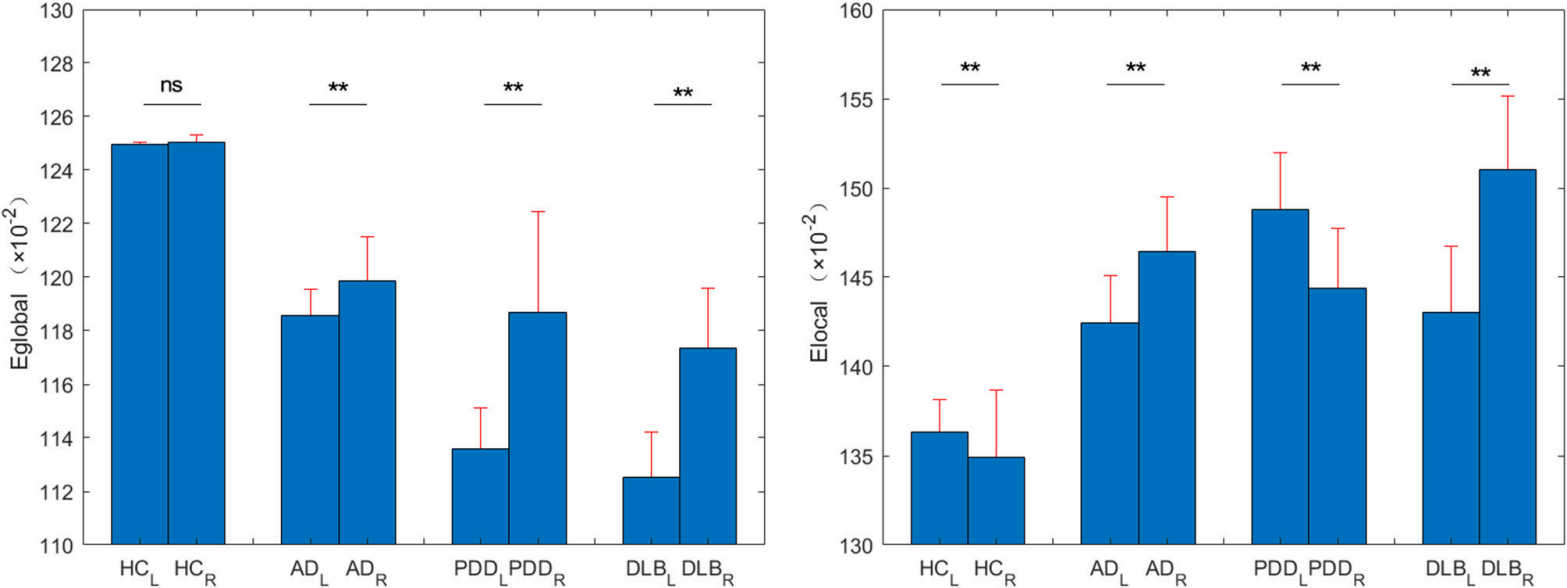
Eglobal and Elocal values for left and right brains from the HC, AD, PDD, and DLB groups. ^**^P < 0.0001.

The within-group asymmetries of global and local network efficiencies for each group are illustrated in Figure 6. The left and right brain global efficiency values for the HC group were 0.6247 and 0.6253, respectively, extremely slight right asymmetry. The right and left brain global efficiency values for the other three groups were 0.5929 vs. 0.5993, 0.5679 vs. 0.5935, and 0.5626 vs. 0.5867, for the AD, PDD, and DLB groups, respectively (*P* < 0.0001); significant rightward asymmetry (i.e., right > left) in global network efficiency was observed in all three groups.

The left and right brain local efficiency values for the HC group were 0.6816 and 0.6744 (*P* < 0.0001), respectively, without either rightward or leftward asymmetry. Analysis of local network asymmetry indicated significant rightward asymmetry in both the AD (0.7122 vs. 0.7323, *P* < 0.0001) and DLB (0.7150 vs. 0.7551, *P* < 0.0001) groups, while significant leftward asymmetry was observed in the PDD group (0.7439 vs. 0.7219, *P* < 0.0001).

### Asymmetry Indices (AI)

**Figure 7:**
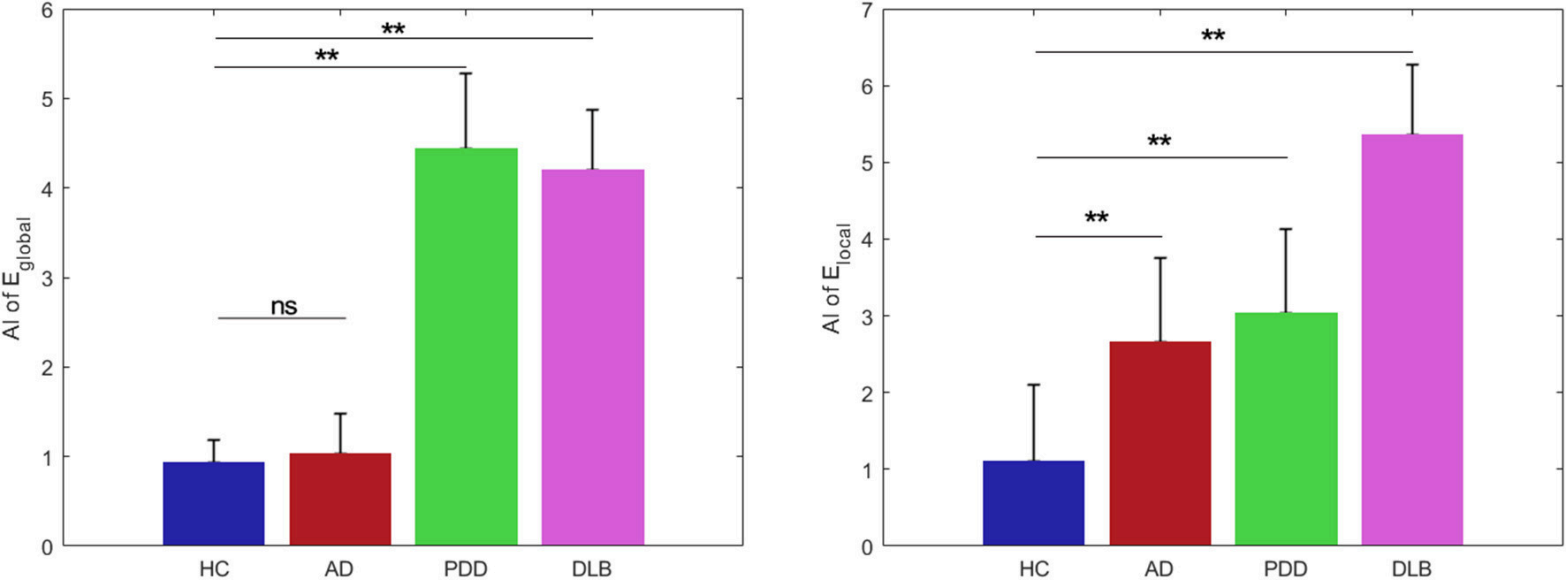
Global efficiency and local efficiency group asymmetry indices. Data are presented as mean ± SD. Blue bars, HC; red bars, AD; green bars, PDD; and magenta bars, DLB groups. ^**^P < 0.0001; ns, no significance.

There were significant differences among the groups in the AI of global network efficiency (*P* < 0.05) (Figure 7). For the HC group, both global and local network AI values approached zero (0.93 and 1.10, respectively), indicating that there was no significant asymmetry in brain collaboration in the control subjects.

*post-hoc* comparisons of the AI values revealed significant differences between those of the HC and patient groups. Compared with the HC group, the PDD and DLB groups exhibited significantly increased asymmetry in global network efficiency (4.44 and 4.20, *P* < 0.0001). Further, there were significant differences in the global network hemispheric topological properties for each hemisphere in the PDD and DLB groups. The AI_global value of the AD group was 0.0011, which was similar to that of the HC group, indicating no obvious asymmetry.

Similarly, the AI_local values of the AD, PD, and DLB groups (2.66, 3.03, and 5.35, respectively) deviated substantially from that of the HC group (1.10) and indicated clear asymmetry. Hence all the dementia groups exhibited results significantly different from those of the HC group (*p* < 0.0001).

## Discussion

This study employed the novel approach of combining brain network and asymmetry analyses to explore the marginal differences in glucose metabolic distributions in brains from patients with three dementia subtypes. The findings of this investigation have potential to facilitate accurate diagnosis and ensure appropriate treatment of patients with various types of dementia.

Briefly, we found that network alterations in the DLB group were broader than those in the AD and PDD group. Also, the three dementia groups exhibited divergent network alterations, manifested as differences in global measures. For example, compared with the HC group, the three dementia groups all showed loss of small-world network characteristics. We also identified different hub regions among the four groups.

Investigation of abnormal hemisphere asymmetry within the groups demonstrated that subjects in the HC group had balanced left and right brains, and the brain network did not exhibit strong bias, either rightward or leftward. Conversely, in the AD and DLB groups, significant rightward asymmetry in local network efficiency was detected, when leftward asymmetry in the hemispheric brain networks of patients with PDD was detected.

Below, we discuss the physiological and pathological implications of these findings.

### Effectiveness of Brain Network Analysis

To verify the effectiveness of employing brain network analysis in this study, we compared our network parameter results with those from previous studies (Table 2). In general, our experimental results are within the scope of those in the existing literature, exhibiting good consistency with previous reports. For example, the C value for the AD group in this study was 0.50, consistent with previous reports; lamdba was 1.03, and previous reports also showed that lamdba values fluctuate around 1.0. Overall, network parameters for the three dementia subtypes included in this study are similar to those in previous reports, indicating that the brain network analysis method used in our investigation are effective, and supporting the validity of comparisons among the three dementia subtypes within a single study using this approach.

**Table 2:** Comparison of network parameters reported in the present study and in previous reports.

**References**	**Experimental image**	**N**	**Disease**	**C**	**L**	**Sigma**	**Lambda**	**Gamma**
**Present Study**	**FDG-PET**	**22**	**HC**	**0.48**	**1.57**	**1.36**	**1**	**1.37**
**Present Study**	**FDG-PET**	**22**	**AD**	**0.59**	**1.64**	**1.28**	**1.04**	**1.33**
Duan et al. (20)	FDG-PET	22	AD	0.6	1.64	1.22	1.03	1.25
Jiang et al. (33)	11C-PiB PET	18	AD	~0.50	~1.59	~1.37	~1.01	~1.38
Yao et al. (34)	MRI	91	AD	~0.6	~1.9	–	~1.1	~1.25
Seo et al. (35)	FDG-PET	216	AD	~0.6	~2	~1.6	–	–
He et al. (36)	MRI	92	AD	~0.4	~1.5	–	~1	~1.2
**Present Study**	**FDG-PET**	**18**	**PDD**	**0.63**	**1.67**	**1.32**	**1.05**	**1.4**
Utianski et al. (37)	EEG(DELTA)	**18**	PDD	–	–	–	1.1	1.3
**Present Study**	**FDG-PET**	**22**	**DLB**	**0.65**	**1.67**	**1.27**	**1.05**	**1.34**
Chen et al. (38)	FDG-PET	22	DLB	0.58	~2.1	1.244	~1.1	~1.4
Peraza et al. (39)	rs-fMRI	22	DLB	0.48	1.8	–	–	1.3

*N, the number of subjects participating in the experiment. The bold values represent the experimental results of our present study in this paper*.

In addition, although the data presented in Figure 2 show that all three dementia groups exhibited small-world network properties, analysis of network parameters revealed that the DLB group experienced more marked changes relative to the other two dementia groups. This may be due to differences in the severity of cognitive loss, and the literature suggests that normal cognitive function is highly dependent on typical functional connectivity (37). The significant loss of small world properties in DLB may be associated with presynaptic dysfunction caused by the α-synuclein aggregates present in the brain cortex, even at early stages of this disease (24, 40). Overall, these results show that brain network analysis is very effective for distinguishing among AD, PDD, and DLB.

### Effectiveness of the Hubs Identified in This Study

In this study, 19, 23, and 20 hub nodes were identified in the AD, PDD, and DLB groups, respectively. These hubs could be considered as biomarkers to aid physicians in distinguishing among dementia subtypes. Comparisons with the literature indicated that the majority of the hubs identified in this study have been reported previously, while the remainder can be explained by physiological and pathological phenomena, further indicating that hubs identified in this study were meaningful.

For example, in the AD group, the hub regions included the left medial frontal gyrus and angular gyrus, which are part of the DMN. DMN activity is established to be abnormal and to result in a disrupted topological structure in AD. In addition, the bilateral hippocampus was included among hub regions in AD. Reduced gray matter volume and abnormal functional connectivity in the hippocampus have also been proven in AD. Currently, the mainstream hypothesis is that AD is associated with pathological accumulation of misfolded proteins, including amyloid-β (Aβ) and tau (41). Hence, one possible explanation for the identified hubs is that they exhibit preferential vulnerability to AD pathology. Alpha-synuclein (α-synuclein) misfolds in the cells of the central nervous system to form Lewy bodies, which accumulate and lead to impairment of nigrostriatal dopamine (DA) neurons.

The results of our analyses of brains from patients with PDD indicated that hubs were mainly concentrated in middle temporal gyrus, middle occipital gyrus, inferior occipital gyrus, and fusiform gyrus. Structural and pathological changes occur in middle occipital gyrus and inferior occipital gyrus in PDD. Voxel-based analysis of FA, using DTI, found that it was significantly reduced in patients with this condition (42). Further, Kim et al. reported that there is significant hypoperfusion in the fusiform gyrus in patients with PDD (29).

Although there is no literature to support significant changes in the middle temporal gyrus of patients with PDD, numerous studies have concluded that this key brain region is closely related to Parkinson's disease (PD) and related disorders. For example, Howlett et al. demonstrated that a combined pathology (comprising Aβ plaques, phospho-tau, and α-synuclein positive features) is a major determining factor in the development of dementia, particularly in the middle temporal gyrus, which contributes to the deterioration of PD to related disorders (43).

In the DLB group, we detected higher betweenness centrality in the right thalamus, consistent with a previous study suggesting that both thalamic nodes have higher node degrees in DLB compared with controls, possibly reflecting compensatory responses (39). Notably, thalamic alterations in DLB appear to be associated with significant attention and cognitive deficits. To a certain extent, this may be attributable to differences in pathological protein deposition (Aβ, α-synuclein, and tau), leading to compensatory responses in the brain network, and ultimately leading to alterations in the hubs of the different dementia groups.

The DLB hubs in the anterior cingulate and paracingulate gyri were first discovered in this investigation. The anterior cingulate cortex (ACC) is part of the brain limbic system and is widely recognized as a structure involved in control-related functions (44). Neuroimaging studies show that separate areas of the ACC are involved in cognition and emotion, and this structure also contributes to emotional and cognitive development. There is a strong correlation between emotional and cognitive impairment in DLB disease manifestations.

### Effectiveness of Seed Correlation Analysis

The results of seed correlation analysis in this study also provide physicians with new insights into means of discriminating among the three dementia subtypes included. Comparisons with the literature also verify the effectiveness of our seed correlation analysis, or provide physiological and/or pathological explanations for our findings.

For example, the results of our seed correlation analysis indicated that, compared with the HC group, regions of strengthened connection in the AD group were primarily located in the brain DMN. These findings can be explained by DMN atrophy, which is generally acknowledged in the context of glucose metabolism (45). Further, many studies have shown that DMN activity is abnormal and develops a disrupted topological structure in AD (41).

In addition, strengthened connections between the bilateral postcentral gyrus and the MTG.R were detected in the PDD group. The postcentral gyrus is the somatic sensory center, and its abnormal function is closely associated with PD, and diffuse glucose anomalies in the postcentral gyrus are correlated with dementia in patients with PD (29–31).

In the DLB group, the connection with the right inferior temporal gyrus was strengthened, consistent with a previous study suggesting that this area is significantly different in patients with DLB relative to controls on medical imaging, and potentially represents either an alternative or adjunctive biomarker, which may reflect compensatory responses (46).

### Effectiveness of Within-Group Asymmetry

The results of within-Group asymmetry analyses provide further evidence of the feasibility of using brain network analysis to discriminate dementia subtypes in the clinic. Through analysis of network parameters (global efficiency and local efficiency), we found that the phenomenon of abnormal hemisphere asymmetry was present in all three groups of patients with dementia. These results support the findings of previous investigations using non-brain network analysis methods. For example, we found that HC subjects exhibited no significant differences between their two hemispheric brain networks, suggesting that the two hemispheres have similar intra-connected pathways in HC. Scholars previously identified rightward asymmetry in AD hemispheric brain networks (16, 26, 47, 48). Notably, the AD patients exhibited significant rightward asymmetry in network efficiency, suggesting that intra-connections in the left hemisphere are less well integrated, with less efficient communication at the hemispheric level, in patients with AD. Our findings are consistent with earlier studies (9, 49).

Similar to the AD group, we also discovered that the DLB group exhibited rightward asymmetry, whereas the PDD group had leftward asymmetry in local efficiency. This may be explained by the fact that PDD progression is accompanied by a decrease in brain motor zone function, since the motor zone is on the left side and the memory area on the right (50).

### Study Limitations

Although the findings of this study indicate that the methods we employed are effective, there are several issues that require further consideration. First, we constructed unweighted and binary networks. During the study, we discarded the direction in which the nodes were connected, and this variable may contain a lot of unknown information, including brain cooperation mode, information transmission, and biological transmitter diffusion mode. Also, partial correlation matrices were used to calculate network parameters and identify altered ROIs. Subsequently, Pearson correlation was applied in this study for ROI-based correlation analysis, which may have introduced bias.

Second, it can be the case that the use of brain templates with better symmetry generate AI values closer to zero; therefore, the development of a symmetrical brain template, with corresponding biological information may facilitate improved exploration of the symmetry of brain function.

Third, the data set included in this study was insufficient. The number of patients included in this study was limited, which may mean that the experimental results are not entirely representative. In future, we plan to collect more disease cases. In addition, the sex of patients in the different groups was not symmetrical, particularly in the DLB group, which is a mental illness more common in men, making it more difficult to collect a sex-balanced sample set for this condition. In future, we plan to further investigate DLB brain network differences between the sexes, to determine whether this characteristic significantly impacts the results.

Finally, in this experiment, the MMSE scale values of the three disease groups did not match exactly, which may have impacted the cognitive function results; however, given the different pathogenesis of the types of dementia studied, it may be difficult to identify perfectly matched subjects.

## Ethics Statement

This study was carried out in accordance with the recommendations of the institutional review board of Huashan Hospital with written informed consent from all subjects. All subjects gave written informed consent in accordance with the Declaration of Helsinki. The protocol was approved by the institutional review board of Huashan Hospital.

## Author Contributions

DC, JL, and HZ made specific experiments. JJ and PW had unique insights in characterization and analysis. DC wrote articles, CZ guided, reviewed and examined the experiments. KS participated in the discussion and gave unique insights.

### Conflict of Interest Statement

The authors declare that the research was conducted in the absence of any commercial or financial relationships that could be construed as a potential conflict of interest.

## Footnotes

^1^https://www.mathworks.com
